# Towards an RNA/Peptides World by the Direct RNA Template Mechanism: The Emergence of Membrane-Stabilizing Peptides in RNA-Based Protocells

**DOI:** 10.3390/life13020523

**Published:** 2023-02-14

**Authors:** Yu Shi, Chunwu Yu, Wentao Ma

**Affiliations:** 1 Hubei Key Laboratory of Cell Homeostasis, College of Life Sciences, Wuhan University, Wuhan 430072, China; 2 College of Computer Sciences, Wuhan University, Wuhan 430072, China

**Keywords:** direct RNA template mechanism, the RNA world, the RNA/peptides world, computer simulation, early evolution, the origin of life

## Abstract

How functional peptides may have arisen is a significant problem for the scenario of the RNA world. An attractive idea, the direct RNA template (DRT) hypothesis, proposes that RNA molecules can bind amino acids specifically and promote the synthesis of corresponding peptides, thereby starting the RNA/peptides world. To investigate the plausibility of this idea, we modeled the emergence of a “membrane-stabilizing peptide” in RNA-based protocells—such a peptide was suggested to have appeared early in the RNA world based on experimental evidence. The computer simulation demonstrated that the protocells containing the “RNA gene” encoding this peptide may spread in the system owing to the peptide’s function. The RNA gene may either originate de novo in protocells or emerge in protocells already containing ribozymes—here we adopt a nucleotide synthetase ribozyme as an example. Furthermore, interestingly, we show that a “nucleotide synthetase peptide” encoded by RNA (also via the DRT mechanism) may substitute the nucleotide synthetase ribozyme in evolution, which may represent how “functional-takeover” in the RNA world could have occurred. Overall, we conclude that the transition from the RNA world towards an RNA/peptides world may well have been mediated by the DRT mechanism. Remarkably, the successful modeling on the emergence of membrane-stabilizing peptide in RNA-based protocells is per se significant, which may imply a “promising” way for peptides to enter the RNA world, especially considering the weak interaction between RNA and the membrane in chemistry.

## 1. Introduction

“The RNA world” hypothesis is popularly adopted to explain the molecular base of primordial life [1,2,3,4]. It assumed that in the beginning, RNA served both as a genetic material and functional material, thus evading the chicken–egg dilemma of “which came first, DNA or proteins?” in the origin of life. Indeed, as we know, some viruses (and viroids) still have an RNA genome. On the other hand, ribozymes were found [5,6] and have now been well studied; most surprisingly, people revealed that the catalytic core of ribosome is RNA instead of proteins [7,8].

Highlighting the importance of heredity, the RNA world hypothesis is often deemed as a representative idea of “replication first” or “genetics first” during the arising of life, and an “opposing” point of view is referred to as “metabolism first” [9]. The viewpoint of “metabolism first” emphasizes that the prebiotic formation of building blocks of life, such as nucleotides, amino acids, and amphiphiles (the membrane components of protocells), should have involved complicated chemistry (not necessarily as modern biochemical pathways) [9,10,11,12,13]. However, in logic, the two classes of these ideas are not mutually exclusive, especially if the “RNA worlders” accept that the building blocks were not ready to form prebiotically. In fact, the RNA world hypothesis needs not assert any things on the chemical pathway towards the prebiotic formation of nucleotides and RNA, and the key point lies in that it offers a concrete explanation on how Darwinian evolution may have begun, because genetic material and functional material are both indispensable for Darwinian evolution [14,15]. Indeed, even those critical viewpoints on the RNA world, for example, the so-called view of “random polypeptide first” [16], have to agree that before the advent of genetic molecules such as RNA, no real Darwinian evolution could have occurred (notably, lacking an information linkage with genetic molecules such as RNA, the random polypeptides could not be deemed to have entered the living world—see the Discussion for a detailed explanation). In other words, obviously, no matter what kinds of chemical processes were developed first during the origin of life, Darwinian evolution must have begun then, thus likely “witnessing” an RNA world: a primordial living world that began to evolve under the rule of natural selection.

If the RNA world was the primordial living world, a subsequent question is how it could have “invented” DNA and proteins, thus ultimately evolving into the modern living world. In the long run, DNA is more stable and should have taken the place of RNA as the major genetic material; proteins are chemically more versatile and should have taken the place of RNA as the major functional material. Then, “after RNA, which came first, DNA or proteins?” This has long been a controversial issue (e.g., [17,18]); both the two possible answers seem to be supported by some evidence or analysis. No matter how, the arising of DNA appears to have been easier, because RNA and DNA can be simply copied into each other via the template-directed synthesis by base-pairing. However, the advent of proteins looks to be much more difficult. In modern life, a complicated “translation machine” is required to synthesis proteins (via deciphering the genetic code), which certainly could not have existed in the beginning. Possibly, in the beginning, there was some simple way (e.g., similar to the template-directed mechanism) to synthesize proteins, or at least short peptides, in the RNA world.

Consequently, a key concern is: “Are there certain ‘straightforward’ specific interactions between RNA sequences and amino acids?”. RNAs binding sites for amino acids were explored via selection-amplification experiments [19,20,21,22]. The results showed that the binding sites tend to bear characteristic sequences, e.g., at least for some amino acids (Arg, His, Ile, Phe, Trp, and Tyr), the RNA-binding-site tends to involve the amino acid’s anticodons/codons [20,21,22]. Interestingly, a statistical research based on RNA–protein interactions in the ribosome showed that anticodons are selectively enriched near their respective amino acid residues [23]. These findings implied that in the RNA world, RNA molecules may have bound amino acids specifically and promoted the synthesis of peptides in a template-directed-like means, i.e., the direct RNA template (DRT) hypothesis [24]. Perhaps later on, the translation system (together with the genetic code) could have been derived from this simple mechanism [20,24,25] (see the Discussion for a more detailed explanation).

**Figure 1:**
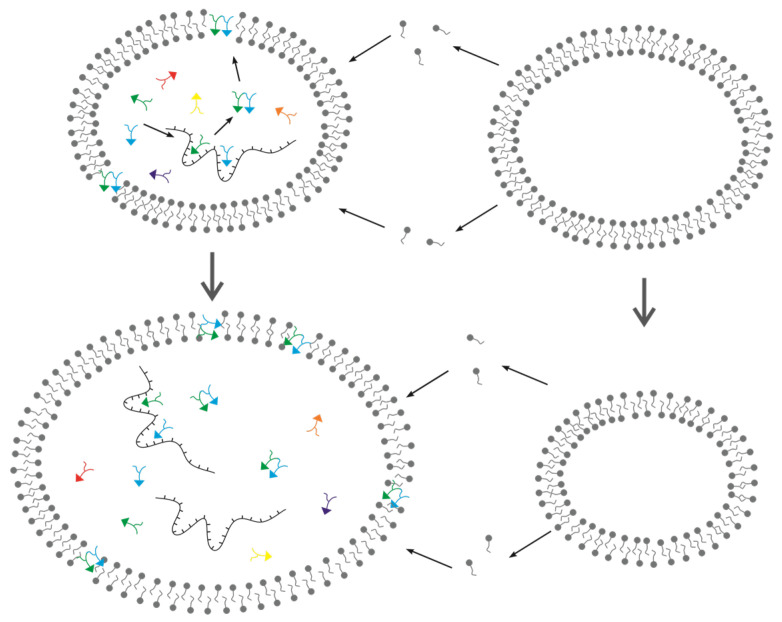
The competition between protocells on account of the effect of MSP (membrane-stabilizing peptide) which is synthesized via the DRT mechanism. The “R-shaped” symbols in different colors represent amino acids of different kinds. The RNA with characteristic sequences binds amino acids specifically and bring them together to form peptide bonds. MSP (here a dipeptide consisting of a blue amino acid and a green one) could enter the membrane and prevent the leaving of amphiphiles (i.e., fatty acids or molecules alike) from the membrane. As a net result of amphiphiles’ interchange between the membrane and the environment, the protocell without MSP would lose amphiphiles and shrink while the protocell containing MSP would grow to a larger scale.

Here, we plan to model the DRT mechanism in order to show whether functional peptides can be introduced into the RNA world by this mechanism. Then, as the modeling target, we must choose a functional peptide which possibly appeared early in the RNA world. Interestingly, an experiment study indicated that a sort of hydrophobic dipeptide could decrease the desorption of fatty acids from the membrane of vesicles, and such a “membrane-stabilizing peptide (MSP)” was suggested to have favored protocells in the competition for limited membrane components [26]. So, if an RNA sequence can bind corresponding amino acids and favor the synthesis of the MSP, the protocells containing this “RNA gene” might “win the survival competition” and spread (become thriving) around the circumstance. Indeed, RNAs polar skeleton makes it difficult to interact with the membrane, so peptides are likely to have been utilized to implement the membrane-associated function that favored the RNA-based protocells. The effect of MSP, as well as the scene of an RNA sequence guiding the peptide’s synthesis by the DRT mechanism (thus, the MSP gene or MSPG), is illustrated in Figure 1.

## 2. Results

### 2.1. The Plausibility Concerning the Spread of the Protocells Containing MSPG

**Figure 2:**
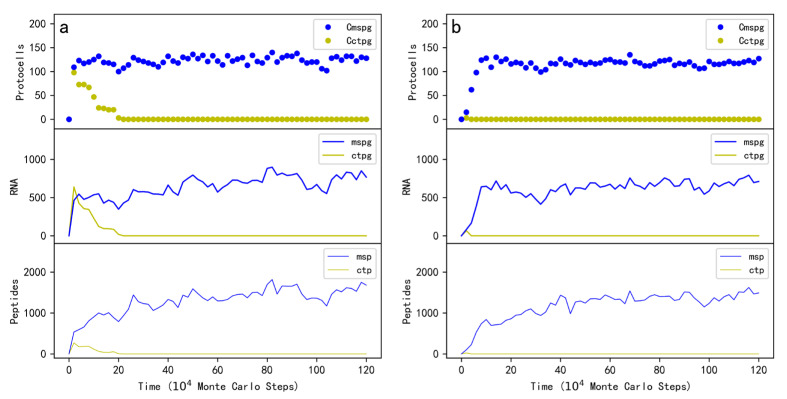
The spread of protocells containing MSPG. Legends: Cmspg—protocells containing MSPG; Cctpg—protocells containing a control peptide’s gene (the peptide has no function); mspg—MSPG; ctpg—the control peptide gene; msp—MSP; ctp—the control peptide. Note that the numbers of peptides and their genes are the ones summed up within the whole system. (**a**) At step 1 × 10^4^, ten protocells each containing five copies of MSPG and five copies of the control peptide gene are inoculated into the system (at locations which are randomly chosen). (**b**) At step 1000, an empty protocell (or say, a vesicle) is inoculated into the system, and by growth and division, the protocells spread in the system. Then, at step 1 × 10^4^, a copy of MSPG is inoculated into one of these empty protocells and a copy of the control peptide gene is inoculated into another empty protocell. This case represents a simulation of the de novo emergence of MSPG via the DRT mechanism in protocells.

First of all, we should make it clear whether the protocells containing MSPG (“MSPG protocells” for short) could spread in the system by virtue of the MSPs function. The model’s system is initialized with amphiphile (fatty acid) precursors, nucleotide precursors, and amino acid precursors (in quantities of *T_APB_*, *T_NPB_*, and *T_AAPB_*, respectively; see Table 1 for descriptions of the parameters). At step 1 × 10^4^, ten protocells each containing five copies of MSPG and the same number of a control RNA species are inoculated into the system. The control RNA species can encode another peptide by the DRT mechanism, but the peptide has no function (thus the RNA species is called “the control peptide gene” here). As time goes on, the MSPG protocells spread in the system (Figure 2a, the top panel). Certainly, the MSPG and the MSP in the system also increase simultaneously (the lower two panels). In fact, by gaining the membrane components continuously, the MSPG protocells would grow larger (refer to Figure 1) and are thus capable of accommodating more gene copies synthesized in the RNA replication; when a protocell reaches a certain scale, it might divide due to the physical instability and the RNA genes within it would be distributed randomly into offspring protocells (see the Methods section for a detailed explanation of the relevant events). Thereby, the MSPG protocells “reproduce” and spread in the system.

Notably, in this case, the control RNA species was introduced together with the MSPG into the same protocells, thus it would also benefit from the MSP synthesized by the MSPG. That is, the control species here also represents a parasite. It can be observed that after a short time of rising together with the MSPG, the control species decreased and finally faded out (Figure 2a, yellow symbols). The reason involves the selection at the protocell level [27,28,29]. As mentioned above, when the protocells grow and ultimately divide, the RNA molecules would be distributed randomly into offspring protocells. Those offspring protocells containing the control species but no MSPG would shrink due to the competition of the membrane components (see Figure 1). Upon the degradation events of protocells and RNA, such as the protocell breaking, the RNA chain breaking, and the RNA chain-end decaying (see Methods), the RNA-based protocells that cannot grow and reproduce would become extinctive. In other words, here we show that in a robust sense (with a consideration of the possible parasite problem), the MSPG protocells can spread in the system.

**Figure 3:**
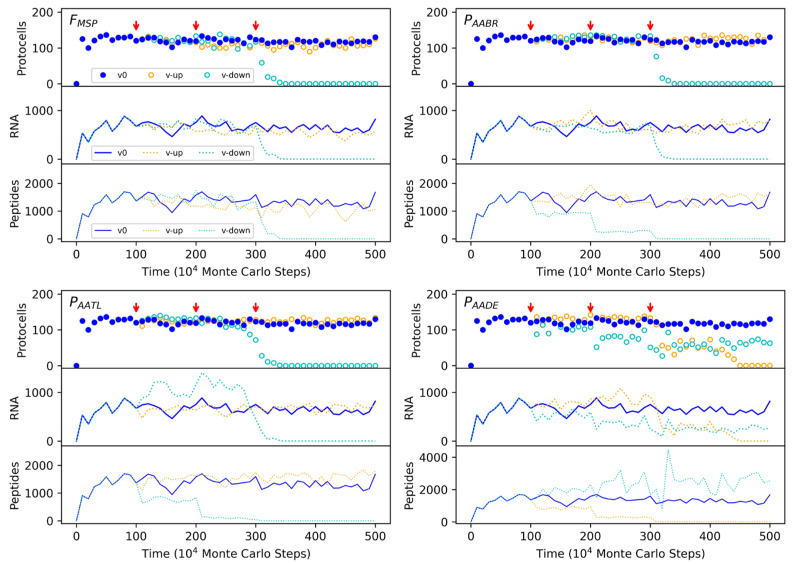
The influences of several key parameters on the spread of the protocells containing MSPG. The filled circles (denoting protocells) and solid lines (denoting RNA or peptides) represent the case adopting the default parameter values (Table 1); actually, it is just the case shown in Figure 2a. The open circles and dotted lines represent the cases in which the value of the parameter naming the subfigure is turned up (orange) or down (cyan) at certain steps (see the red arrows). Legends: v0—the case adopting the default value; v-up—the case of value turning up; v-down—the case of value turning down. The legends apply to the whole figure. ***F_MSP_***, with a default value of 1, when turned up, is changed to 5, 20, and 100 at the three turning steps, respectively, and when turned down, is changed to 0.2, 0.05, and 0.01 at the three turning steps, respectively. ***P_AABR_***, with a default value of 0.9, when turned up, is changed to 0.95, 0.98, and 0.99, and when turned down, is changed to 0.2, 0.05, and 0.01. ***P_AATL_***, with a default value of 0.5, when turned up, is changed to 0.9, 0.95, and 0.98, and when turned down, is changed to 0.05, 0.005, and 5 × 10^−4^. ***P_AADE_***, with a default value of 0.1, when turned up, is changed to 0.2, 0.5, and 0.9, and when turned down, is changed to 0.05, 0.02, and 0.01.

In order to confirm that the spread of MSPG protocells is indeed attributed to the function of MSP, we conducted some key parameter analysis. First, when the factor concerning MSP (*F_MSP_*, see the Methods for an explanation on how this factor works) is turned down to some extent, the MSPG protocells would decline and finally disappear (Figure 3). That is, when the MSP within the membrane becomes inefficient to prevent the leaving of fatty acids from the membrane, the spread of MSPG protocells may no longer be sustained. Next, when the DRT mechanism becomes inefficient, thus MSP is difficult to synthesize, the MSPG protocells would also fade out. For example, when the rate of amino acids binding onto RNA (*P_AABR_*) or the rate of amino acids linking into peptides on an RNA template (*P_AATL_*) is turned down to some degree, the MSPG protocells descend correspondingly (Figure 3).

Then, interestingly, either too high or too low a probability concerning the degradation of peptides (*P_AADE_*) would disfavor the spread of MSPG protocells (Figure 3). It is easy to understand that a high degradation rate of peptides may be unfavorable because the lifespan of MSP may be too short to functionally support the spread of MSPG protocells. However, why would a too low degradation rate of peptides also be unfavorable? In fact, the reason is associated with the “role separation” between genes and functional molecules in this scene. With the “cell division”, because of the random assortment of MSP and MSPG between offspring protocells, some protocells which do not contain MSPG may still reserve some MSP within their membrane. Then, if the degradation of MSP is too slow, the MSPG protocells would not manifest their superiority. Indeed, for a lower degradation rate, the level of MSP would be higher (see the lowest panel in Figure 3-*P_AADE_*, the cyan dotted line; note that here the vertical coordinate adopts a larger scale than the corresponding panels in the other subfigures), but both the MSPG protocells and MSPG in the system would decline (the upper two panels in Figure 3-*P_AADE_*, cyan symbols). This phenomenon should represent a significant effect associated with the labor division between genetic and functional molecules, which may be named as “function-lagging”.

Besides these key parameters, we also conducted an analysis of the other parameters (Figure S1 and S2, Table S1). See Box S1 for a comment on their influence. Notably, similarly to *P_AADE_* (the probability of amino acid residue decaying at the end of a peptide), *P_PBB_* (the probability of peptide bond breaking) is also a parameter associated with the degradation of MSP. However, the parameter analysis showed that the turning down of P_PBB_ has little influence on the spread of the MSPG protocells. That is, the so-called “function-lagging” effect seems to have not occurred. We suspected that it is because the default value of *P_PBB_* adopted here (0.01) is significantly lower than that of *P_AADE_* (0.1). Then, here the “bottle-neck” concerning the degradation of MSP is *P_AADE_*; when P_PBB_ is turned down further, little influence could be observed. To verify this speculation, we interchanged the default values of the two parameters, i.e., *P_PBB_* = 0.1 and *P_AADE_* = 0.01, and ran the analysis again. Indeed, this time it is the turning down of *P_PBB_* (instead of *P_AADE_*) that shows the “function-lagging” effect (Figure S3-*P_AADE_’* and *P_PBB_’*, cyan symbols). Note that here the difference between the two parameters could be reflected by the turning up analysis: too high a *P_AADE_* would be obviously unfavorable (Figure S3-*P_AADE_*, orange symbols), but this seems to be not so obvious for *P_PBB_*—that is, MSP declines obviously, but MSPG and the MSPG protocells are still sustained (Figure S3-*P_PBB_*’, orange symbols). The reason should be that when the peptide bond of MSP breaks on account of a high *P_PBB_*, the resulting amino acids could be used directly to produce the MSP again, whereas when an amino acid residue decays at the end of an MSP on account of a high *P_AADE_*, the resulting amino acid precursor could not be exploited directly.

### 2.2. Modeling the De Novo Emergence of MSPG in Protocells

Above we have shown that the protocells containing MSPG may spread in the system owing to the function of MSG. This already suggests that the MSPG may have emerged de novo in RNA-based protocells. However, to avoid the influence of degradation events by accident, we inoculated initially into the system some protocells each containing a few copies of MSPG (ten protocells each with five copies of MSPG for the case shown in Figure 2a). In the reality of the life’s origin, the simultaneous appearance of these protocells and RNA genes was impossible. No matter how, to be more convincing, a “straightforward simulation” on the de novo emergence was conducted; indeed, when inoculating one MSPG molecule into one “empty protocell”, we witnessed the subsequent thriving of MSPG protocells in the system (Figure 2b). Note that in this case, the control peptide gene was inoculated into another empty protocell, i.e., it could not act as a “parasite” (unlike the case shown in Figure 2a), and hence the protocells containing the control peptide gene, as well as the gene and corresponding peptide, never rose (Figure 2b, yellow symbols).

### 2.3. The Plausibility Concerning the Spread of Protocells Containing Both MSPG and a Functional Ribozyme

Then, it is interesting to see whether the MSPG, as an RNA gene encoding a functional peptide, could cooperate with a functional ribozyme (which is the gene of itself). The RNA template-directed polymerase ribozyme (also known as “RNA replicase”, REP for short) has long been suggested to be a candidate for the ribozyme emerging in an early RNA world [2,30,31,32] and there have already been many experimental efforts to construct such a ribozyme (e.g., [33,34,35,36]) (though these have not yet been achieved). Alternatively, a ribozyme capable of catalyzing the synthesis of RNAs building block [2], i.e., a nucleotide synthetase ribozyme (NSR), can also favor its own replication by supplying the monomers and thus may (in principle of Darwinian evolution) as well be a candidate [37] (there was also experimental work concerning NSR [38,39,40]). In fact, the spread of one of the two ribozymes or both of them (thus representing their cooperation) has been demonstrated in computer modeling work [37,41,42,43,44,45]. Here, on adopting one ribozyme to see if the MSPG could cooperate with, we tend to choose NSR; if there was ever a stage of protocells containing only one ribozyme, it is more likely to have been the stage of NSR protocells, because the raw material for the NSR protocells could be the precursors of nucleotides, which should have been much easier to permeable through the protocells’ membrane than the nucleotides per se [46,47], which were required by the REP protocells.

**Figure 4:**
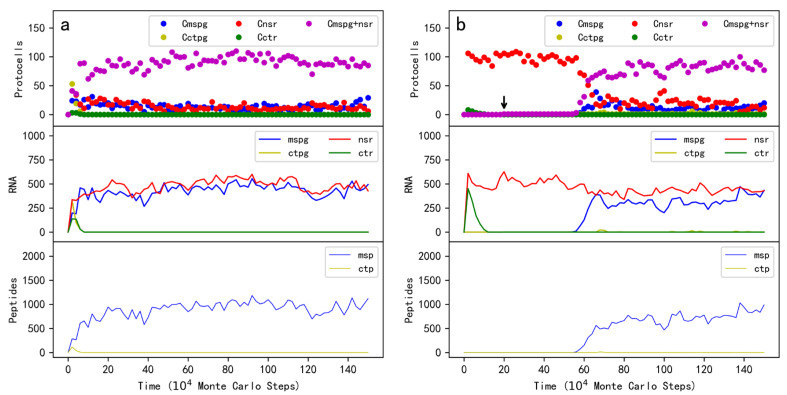
The spread of protocells containing both MSPG and NSR (nucleotide synthetase ribozyme). Legends in addition to those in Figure 2: Cnsr—protocells containing NSR; Cctr—protocells containing a control RNA (with no function and cannot encode peptides); Cmspg + nsr—protocells containing both MSPG and NSR; nsr—NSR; ctr—the control RNA. (**a**) At step 1 × 10^4^, ten protocells each containing five copies of MSPG, a control peptide gene, NSR and a control RNA are inoculated into the system. (**b**) At step 1 × 10^4^, ten protocells each containing five copies of NSR and a control RNA are inoculated into the system. Then, at step 2 × 10^5^ (see the black arrow) and thereafter, every 1 × 10^4^ steps, a copy of MSPG is inoculated into one of the NSR protocells and a copy of a control peptide gene is inoculated into another NSR protocell. The case (*b*) represents a simulation of the emergence of a peptide’s RNA gene (MSPG)—based on the DRT mechanism—in protocells already containing a ribozyme (NSR).

To investigate the plausibility concerning the spread of protocells containing both MSPG and NSR, after the initial introduction of raw materials, at step 1×10^4^, ten protocells each containing five copies of MSPG, NSR, and two control RNA species, are inoculated into the system. Wherein, one control species encodes a non-functional peptide by the DRT mechanism, thus as a control of MSPG, and another control species does not encode any peptides, thus as a control of NSR. As time goes on, the protocells containing both the MSPG and NSR spread (Figure 4a, the top panel). Certainly, MSPG and NSR increase in the system simultaneously (the middle panel); MSP also increases (the bottom panel). Notably, the two control RNA species was introduced together with MSPG and NSR into the same protocells, but they cannot spread. In other words, similar to the case of the MSPG protocells (Section 2.1, Figure 2a), here we show that in a robust sense (considering possible parasite problems), the MSPG-NSR protocells can spread in the system. In addition, significantly, here we see that neither the protocells containing only MSPG or the protocells containing only NSR cannot spread (blue circles and red circles, respectively), demonstrating the importance of a cooperation between the MSPG and NSR.

### 2.4. Modeling the Emergence of MSPG in Protocells Containing Ribozymes

In Section 2.2, we have shown that MSPG may have emerged de novo from protocells containing no functional ribozymes. However, the scenario regarding the emergence of peptides within the RNA world might be more interested in whether functional peptides encoded by RNA genes could have emerged from a “ribozyme world”. After confirming the plausibility that the protocells containing both MSPG and NSR can spread in the system, we may expect that if MSPG could appear within a protocell already containing NSR, the MSPG-NSR protocells would spread. However, in the simulation, we found that, different from the case concerning the de novo emergence of MSPG in the protocells (Figure 2b), the inoculation of one copy of MSPG into one NSR protocell is much more difficult to bring about the anticipated spread. The reason should be that here the nucleotide precursors are nearly exhausted due to the spread of the NSR; in this scene, the materials for further RNA replication would come from other RNA molecules’ degradation. That is, the MSPG may have degraded before it could reach a thriving level via replication. We noticed that this just represents the situation in reality: after the initiation spread of early species in the origin of life, the resources should have been nearly exhausted, and the superior species would have sustained in sacrifice of the others, which degraded to replenish the resources (that is, the earliest survival competition).

However, in the real history of a “huge” time scale concerning the origin of life, those beneficial traits may have appeared repeatedly. To model this situation, in a system with the spread of the NSR protocells, we inoculated one copy of MSPG into one of the NSR-protocells at intervals (every 1 × 10^4^ steps; the NSR-protocell was chosen randomly for each time of the inoculation). Then, we saw that after one of these inoculations, the MSPG-NSR protocells began to rise and ultimately substituted the NSR protocells to become the major protocells in the system (Figure 4b). Note that a control peptide gene was also inoculated at the intervals, but the control peptide gene, as well as the protocells containing it, could not thrive (Figure 4b, yellow symbols).

### 2.5. The Plausibility Concerning the Spread of Protocells Containing Both MSPG and Another Functional Peptide’s RNA Gene

**Figure 5:**
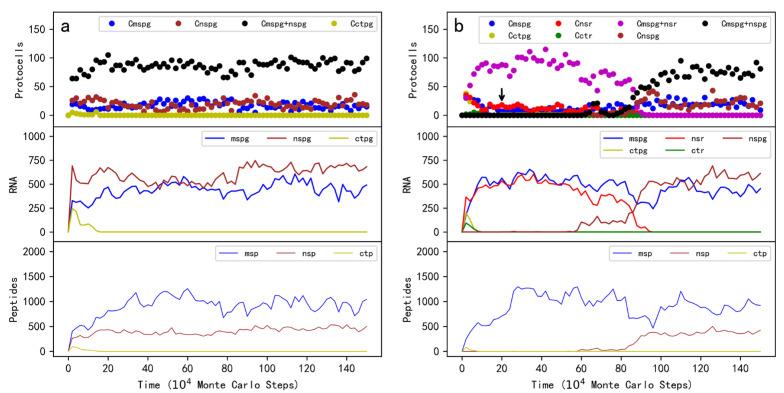
The spread of protocells containing both MSPG and NSPG (nucleotide synthetase peptide gene). Legends in addition to those in Figure 2 and Figure 4: Cnspg—protocells containing NSPG; Cmspg + nspg—protocells containing both MSPG and NSPG; nspg—NSPG; nsp—NSP. (**a**) At step 1 × 10^4^, ten protocells each containing five copies of MSPG, NSPG, and a control peptide gene are inoculated into the system. (**b**) At step 1 × 10^4^, ten protocells each containing five copies of MSPG, a control peptide gene, NSR, and a control RNA are inoculated into the system. Then, at step 2 × 10^5^ (see the black arrow) and thereafter, every 1 × 10^4^ steps, a copy of NSPG is inoculated into one of the MSPG-NSR protocells and a copy of a control peptide gene is inoculated into another MSPG-NSR protocell. *P_NF_* = 0.01, *P_NFR_* = 0.2, and *P_NFP_* = 0.9 (other parameters adopt the default values, Table 1). The case (*b*) represents a simulation of the functional takeover of a ribozyme (NSR) by an RNA gene that encodes a peptide with the same function (NSPG)—based on the DRT mechanism—in protocells.

Beyond the issue concerning the cooperation of the MSPG and functional ribozymes, another attractive issue is whether the MSPG could co-spread with a different RNA gene which also encodes a functional peptide. Then, we assumed another peptide that can catalyze the synthesis of nucleotides named “nucleotide synthetase peptide (NSP)”. The corresponding RNA gene is called “nucleotide synthetase peptide gene (NSPG)”. Indeed, after the initial inoculation of protocells containing MSPG, NSPG, and a control peptide gene, the MSPG-NSPG protocells spread in the system, whereas neither the protocells containing the control peptide gene, nor those containing solely the MSPG or the NSPG, could spread (Figure 5a).

### 2.6. Modeling the Takeover of Ribozyme by the RNA Gene Encoding a Peptide with the Same Function in Protocells

Above we have shown that MSPG can either cooperate with NSR or NSPG. Then, it becomes attractive to see whether the supposed “functional takeover” of ribozymes by peptides/proteins (or more precisely, the corresponding RNA genes) could occur. So, in a system with the spread of the MSPG-NSR protocells, we inoculated one copy of NSPG into one of these protocells intermittently (every 1 × 10^4^ steps; the MSPG-NSR protocell was chosen randomly for each time of the inoculation). Then, we saw that after one of these inoculations, the MSPG-NSPG protocells began to thrive and ultimately substituted the MSPG-NSR protocells to become the major protocells in the system (Figure 5b). Note that while a control peptide gene was also inoculated at the intervals, the control peptide gene, as well as the protocell containing it, could not spread (Figure 5b, yellow symbols).

Here, we noticed that it seems that NSR would fade out because it is less efficient than NSPG, which encodes the same function. Then, we were curious about whether the two “competing” species could co-spread if they have an equivalent efficiency. So, we inoculated protocells containing both the NSR and NSPG into the system in the beginning and attempted to achieve the assumed equivalent efficiency by an adjustment of the parameters. In the model, the catalytic efficiency of NSR is denoted by *P_NFR_*, and that of NSP is denoted by *P_NFP_* (see Table 1). However, note that the ultimate efficiency of NSR and NSPG is not simply represented by the two parameters because different from NSR, which performs its function by itself, NSPG functions by encoding the peptide NSP (thus involving different mechanisms and factors). Indeed, for instance, when *P_NFR_* = 0.5 and *P_NFP_* = 0.3, it is NSPG that wins the competition and spread (Figure S4a). Based on this case, while keeping *P_NFR_* constant, we turned down *P_NFP_* to explore the assumed balance. We found that only a small drop in this efficiency, i.e., *P_NFP_* = 0.2, would reverse the result; that is, NSR wins (Figure S4b). Further on, we turned up *P_NFR_* in a finer scale: in the case of *P_NFP_* = 0.25, NSR still wins (Figure S4c), but for *P_NFP_* = 0.275, NSPG wins (Figure S4d). In other words, even if there might be a balance between the two functional species, here *P_NFP_* should adopt a value as sensitive as between 0.25 and 0.275, which means they are actually difficult to co-spread. Corresponding to reality during the origin of life, we can envision that a ribozyme would have dominated till a peptide gene encoding the same function (a more efficient one in general) took its place; the ribozyme would then disappear.

## 3. Discussion

In this study, firstly we looked into the plausibility concerning whether the protocells containing an RNA species (MSPG) “encoding” the membrane-stabilizing peptide (MSP) via the DRT mechanism can spread in the system (Figure 2a). After confirming this plausibility, we modeled the de novo emergence of MSPG in the protocells (Figure 2b). Next, we looked into the plausibility of the cooperation of MSPG with a ribozyme (nucleotide synthetase ribozyme, NSR) in protocells (Figure 4a). After confirming this plausibility, we modeled the arising of MSPG within the protocells containing NSR (Figure 4b), which means that MSPG could also have emerged in protocells already containing ribozymes. Finally, we showed the plausibility of the cooperation of MSPG with another functional peptide gene (nucleotide synthetase peptide gene, NSPG, also encoding the corresponding peptide by the DRT mechanism) in protocells (Figure 5a). Based on this plausibility, we modeled the arising of NSPG from the protocells containing MSPG and NSR (i.e., the functional takeover of NSR by NSPG) (Figure 5b), which implies a pathway from the RNA world to a more efficient RNA/peptides world.

In the supposed RNA world, RNA acted as both genetic and functional molecules. However, to walk out of the world, the labor division between genetic and functional molecules was inevitable. There were previous studies modeling the labor division on account of the emergence of DNA in the RNA world, in which genes were carried on DNA and functions were carried out by ribozymes [48,49]. Here, we model the labor division on account of the emergence of peptides in the RNA world, in which genes were carried on RNA and functions were carried out by peptides. Interestingly, in our parameter analysis, we observed a phenomenon that should be attributed to the labor division, the “function-lagging” effect (Figure 3-*P_AADE_*; Figure S3 for an “enhanced” analysis). Indeed, upon the “cell division”, due to the random assortment of peptides and genes between offspring protocells, the functional peptides and genes would not necessarily always accompany each other. Then, if the degradation of the functional peptide is very slow, those protocells containing the peptide but not the peptide’s gene would continue to benefit from the peptide’s function for a long time. That is, the significance of the peptide’s gene would be weakened, and the spread of the protocells containing the gene would be unfavored. In other words, with the labor division between the genetic and functional molecules, a functional molecule should be somewhat easy to degrade to “manifest” the significance of the corresponding gene, which can direct the production of the functional molecule repeatedly. This seems to be also the case in the modern living world.

As already mentioned, for the emergence of DNA in the RNA world, there was a straightforward chemical mechanism to transfer information, i.e., the template-directed copying—either from RNA to DNA or vice versa. The direct RNA template (DRT) hypothesis suggests a potential comparable mechanism for the emergence of peptides in the RNA world [20,22,24]. In particular, the experimental work has suggested that even dipeptides may have been obviously functional for protocells, not only referring to the MSP, but also to a dipeptide which can catalyze the formation of the MSP [26]. That is to say, given the DRT mechanism, functional peptides “encoded” by RNA genes may have been ready to emerge in the RNA world.

Indeed, the greatest attraction of the DRT mechanism lies in that it formulates a simple but effective way to transfer information from RNA into peptides. In the mechanism, only RNA with specific subsequences can bind corresponding amino acids and ultimately favor the production of a specific peptide. Since the famous Miller–Urey experiment [50] and many other following studies, we have known that amino acids may have formed readily in the prebiotic environment (owing to their chemical simplicity, they are much easier to form than nucleotides). The random ligation of the amino acids should have produced peptides. However, the peptides would not “enter the living world” until they could be encoded by genetic material. For instance, if a peptide favored a protocell containing it, but the further formation of the peptide still rested on the random ligation of amino acids (i.e., no heredity), for the protocell, the peptide would be no better than an environmental factor such as water or ions. In other words, a real RNA/peptides world should mean a “living world”, with RNA and peptides linked by information rather than simply a “chemical world” containing both RNA and peptides as materials [14,15].

Another attractive point of the DRT hypothesis is that it implies a potential way for the arising of the genetic code. The relevant experiments showed that a characteristic RNA sequence binding a specific amino acid tends to contain the anticodons/codons (especially the anticodons) of the amino acid [20,21,22]. Simultaneously, there is evidence showing that anticodons are enriched adjacent to corresponding amino acid residues within the structure of the RNA–protein complex (the ribosome) in modern cells [23]. Based on such a specific relationship, the DRT hypothesis itself can envision an “abstract scenario” on the origin of the genetic code [20,24]. As a more concrete scenario, in one of our previous papers, we suggested that a proto-translation system, including proto-tRNAs, proto-rRNAs, and proto-mRNAs may have derived from the DRT mechanism “on request of” saving resources for genes’ replication, i.e., the DRT-RP hypothesis (RP for “replication parsimony”) [25]. For example, to encode a dipeptide, for the extant translation mechanism, a gene sequence of six nucleotides in length is sufficient, whereas for a DRT mechanism, it is likely that more than twenty nucleotide residues are required [20,21,22] (to avoid intensive computation, in the present study we assume that the RNA template for the synthesis of a dipeptide is ten nucleotides in length, five for each amino acid). Indeed, for longer peptides or proteins, the DRT mechanism would have been too “wasteful” in coding and should have ultimately been taken over by a proto-translation system (see [25] for details).

Remarkably, here we successfully modeled the takeover of a ribozyme (NSR) by an RNA gene encoding a peptide with the same function (NSPG) (Figure 5b). It seems that the NSR and NSPG are difficult to co-spread; that is, only one of them could spread, resting with the relative efficiency between them (Figure S4). Certainly, being more versatile in chemistry, peptides/proteins would, sooner or later, have taken the place of RNA as a major functional material in the living world. This result of “no co-spread” is consistent with the story from the RNA world to the modern living world—that is, apart from some key functional roles such as that in the ribosome, few “relics” from the RNA world have been found.

As the major modeling target in this study, the membrane-stabilizing peptide was experimentally evidenced to favor the growth of vesicles containing it [26]. In the paper reporting this experimental work, the authors even implied that some ribozyme could catalyze the formation of the peptide and thus have initiated Darwinian evolution. However, considering the heredity of the peptide’s information (as explained above), the ribozyme mentioned by the authors should have been an RNA gene encoding the peptide instead. Given this modification in notion, we successfully modeled the scene concerning such an initiation of Darwinian evolution (Figure 2b). In other words, the peptide encoded by RNA may have emerge de novo, before the emergence of any potential ribozymes. Interestingly, if this was indeed the case in real history, it would also be reasonable to question the existence of an RNA world in which many ribozymes were thriving; perhaps in the initial stage of life, it was just peptides that played the major functional roles (or say, never a pure RNA world). Accompanying with the arising of a proto-translation system, more long functional peptides/proteins emerged. Then, maybe the most important ribozymes were just the proto-rRNA which catalyzed the formation of peptide bonds. Indeed, to date, no ribozyme as supposed to have ever been significant in the beginning of the RNA world, such as the RNA replicase (REP) or the nucleotide synthetase ribozyme (NSR), has been constructed successfully in the lab in a full sense.

## 4. Methods

### 4.1. The Model

We conducted the computer simulation using a Monte Carlo method similar to that used in our previous work concerning the evolution of the RNA world (refer to [37,42,43,45,49,51,52,53]). The modeled system is a two-dimensional one with an *N* × *N* square grid. Molecules are distributed within the grid rooms, including nucleotides, RNA, amphiphiles (i.e., fatty acids or molecules alike, as membrane components), amino acids, and peptides, as well as the precursors of nucleotides, amphiphiles, and amino acids, respectively. Amphiphiles may assemble at the boundary of a grid room and form a membrane, then the grid room is occupied by a protocell.

**Figure 6:**
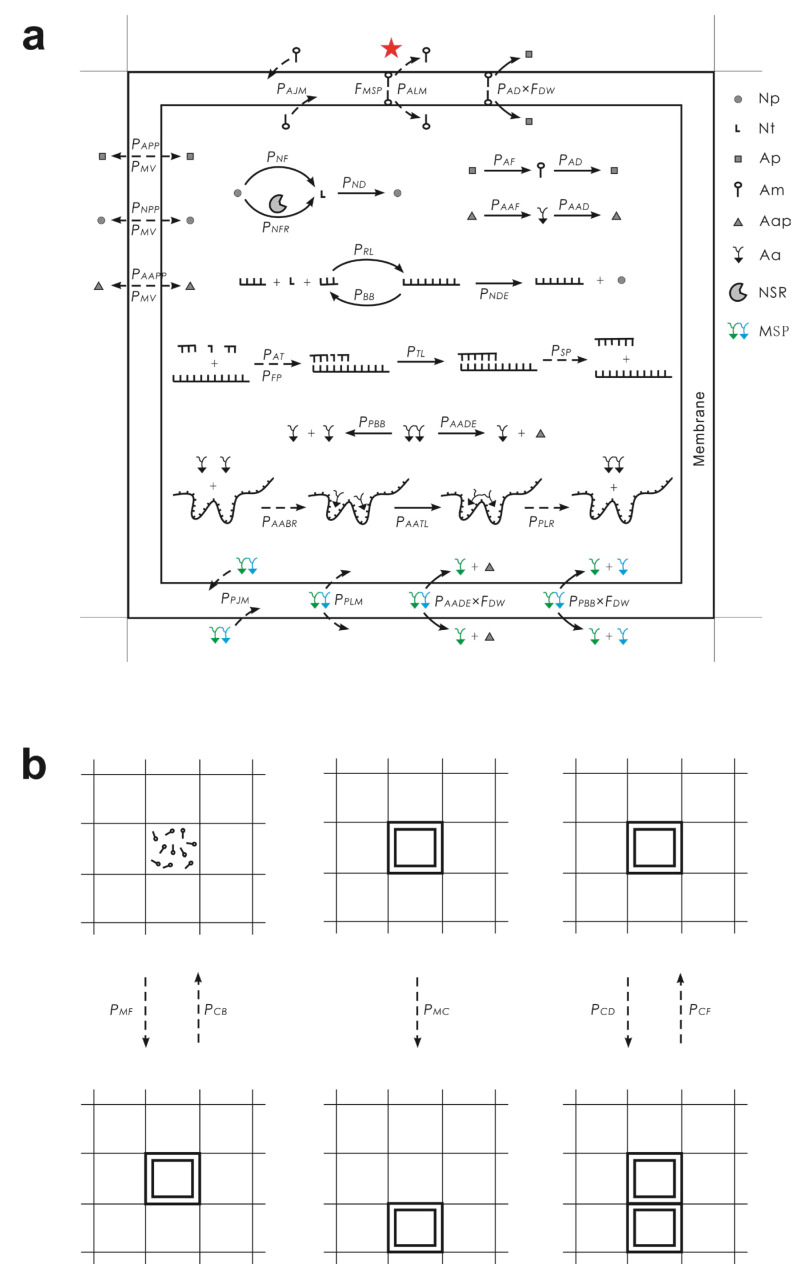
Events occurring in the model system and associated probabilities. Solid arrows denote chemical reactions and dashed arrows represent other events. Legends: Np—nucleotide precursor; Nt—nucleotide; Ap—amphiphile precursor; Am—amphiphile; Aap—amino acid precursor; Aa—amino acid; NSR—nucleotide synthetase ribozyme; MSP—membrane-stabilizing peptide. The events occurring within a protocell are shown in (**a**). The events concerning the behaviors of the protocells are depicted in (**b**), which adopts a smaller scale. For a naked room, there would be no membrane and associated events. Note that the inverted “R-shaped” symbol in black represents an amino acid in general (any possible kinds), whereas the amino acids constituting MSP are draw in color to highlight the events occurring specially to the peptide. The function of MSP is manifested as its influence on the leaving of amphiphiles from the membrane (*P_ALM_*) by the factor *F_MSP_* (indicated by the red star; see the text for a detailed explanation). For the cases modeling NSPG (nucleotide synthetase peptide gene), the formation of a nucleotide may be catalyzed by NSP (another specific dipeptide) with the probability *P_NFP_* (not depicted in this figure).

In each time step (Monte Carlo step), certain events may occur to molecules and protocells with defined probabilities (see Figure 6 for a schematic of the events occurring in the system and their associated probabilities; see Table 1 for descriptions of the probabilities). Only the molecules within the same grid room may interact with each other. The possible events occurring for a molecule include its movement to an adjacent room (related probability: *P_MV_*). A protocell may also move to an adjacent room (*P_MC_*) (while it pushes away molecules in that room).

Nucleotide precursors may form nucleotides (randomly as A, G, C, or U) (*P_NF_*), amphiphile precursors may form amphiphiles (*P_AF_*), and amino acid precursors may form amino acids (randomly as P, Q, R, S, T, or L, i.e., the assumed six types of amino acids in the system, representing some simple amino acids existing in the prebiotic environment) (*P_AAF_*). Nucleotides, amphiphiles, and amino acids may also decay into their precursors (*P_ND_*, *P_AD_*, and *P_AAD_*, respectively).

Nucleotides may join to form RNA via random ligation (*P_RL_*). An RNA molecule may attract substrates (nucleotides or oligomers) (*P_AT_*) via base-pairing, with certain fidelity (*P_FP_*) and substrates aligned on the template may be ligated (*P_TL_*), that is, the template-directed synthesis. The substrates or the full complementary chain may separate from the template (*P_SP_*). Phosphodiester bonds within an RNA chain may break (*P_BB_*) and thus the RNA molecule turns into two fragments. A nucleotide residue at the end of an RNA chain may decay into a nucleotide precursor (*P_NDE_*). An RNA containing a characteristic domain is assumed to be able to catalyze the synthesis of nucleotides (thus act as a nucleotide synthetase ribozyme, NSR) (*P_NFR_*).

Amphiphiles with a sufficient number (larger than *L_AM_*) may accumulate at the boundary of a grid room and form a membrane (*P_MF_*), thus “creating” a protocell. Amphiphiles may join or leave the membrane (*P_AJM_* and *P_ALM_*, respectively). Nucleotide precursors, amphiphile precursors, and amino acid precursors may permeate through the membrane (*P_NPP_*, *P_APP_*, and *P_AAPP_*, respectively). Nucleotides, RNA, amino acids, and peptides are assumed to be unable to permeate through the membrane. A protocell may fuse with another protocell in an adjacent grid room (*P_CF_*), divide (with an offspring protocell occupying an adjacent grid room) (*P_CD_*), and break (*P_CB_*), resulting in the disassembly of the amphiphiles.

The major events described above were introduced as we modeled the RNA-based protocells previously [43,45,49,51,52]. Considering that the model is already quite complicated, when introducing amino acids, peptides, and the DRT mechanism here, we adopted three major simplifications. First, peptides longer than dipeptides are not considered. This simplification is based on the experimental work which serves as a basis of the present modeling; dipeptides could act as the membrane-stabilizing peptide and may also conduct certain catalysis [26] (note that here we have assumed a nucleotide synthetase peptide, i.e., NSP, as another functional dipeptide). Second, the random ligation between amino acids is not considered (actually the random ligation should be much less efficient than the DRT ligation; as a reference, the random ligation between nucleotides is here also quite inefficient, i.e., the default value of *P_RL_* = 1 × 10^−6^). Third, as for RNA binding amino acids or peptides, we assume that it would adopt a folding conformation and would not experience normal events for free RNA, that is, it could not attract nucleotides/oligonucleotides, would not degrade (due to the protection by amino acids/peptides), and would not conduct the random ligation with other RNA/nucleotides (we may envision that the chain end is concealed within the folding structure). Then, the new events introduced here are described as follows.

An amino acid may bind specifically to an RNA site with a characteristic sequence (*P_AABR_*) and two amino acids aligned adjacent on an RNA template may be ligated (*P_AATL_*), that is, the DRT mechanism. The peptide (or amino acids) may leave the RNA template (*P_PLR_*). The peptide bond may break (*P_PBB_*). The amino acid residue at the end of a peptide may decay (*P_AADE_*); a dipeptide would then turn into an amino acid and an amino acid precursor. Of all the possible dipeptides, only the membrane-stabilizing peptide can join the membrane (*P_PJM_*). The MSP may also leave the membrane (*P_PLM_*). When modeling the functional takeover of ribozyme by peptides, another kind of dipeptide was assumed to act as an NSP (nucleotide synthetase peptide) (*P_NFP_*).

Notably, similar to our previous modeling work concerning the evolution of the RNA world, the energy problem is here not considered explicitly. Nucleotides and amino acids are implicitly assumed to be activated. In particular, when nucleotides or amino acids form from the degradation of RNA or peptides, they are assumed to be activated again immediately to be exploited in the further synthesis of RNA or peptides. In reality, this may have involved chemical energy in the hatchery of the primordial life, such as hydrothermal vents at the sea bottom [54,55,56] or hydrothermal fields on land [57,58], as supposed. The focus here is that given a sufficient energy source, whether peptides could have emerged in RNA-based protocells by the DRT mechanism, in other words, the issue addressed in the simulation, concerns the plausibility of evolution instead of the chemical aspect. In addition, on the chemical aspect, the source of materials is here simplified (also similar to our previous work), abstractly represented as nucleotide precursors, amino acid precursors, and amphiphile precursors. In reality, the formation of them should have involved prebiotic synthesis in the hatchery of the primordial life.

Worth mentioning at this time is that the protocells in the model system are competing for materials but not energy because the nucleotides and amino acids are assumed to be always “activated”, whereas the total materials in the system, including those concerning RNA, peptides, and the membrane of protocells are assumed to be limited (related to *T_NPB_*, *T_APB_*, and *T_AAPB_*); in reality, competitions for materials and energy are both possible in Darwinian evolution.

### 4.2. The Setting of Parameters

The parameters should be set according to some rules. Reactions catalyzed by ribozymes should be much more efficient than corresponding non-enzymatic reactions, so *P_NFR_* >> *P_NF_* and *P_NFP_* >> *P_NF_*. Template-directed ligation should be much more efficient than “random ligation”, so *P_TL_* >> *P_RL_*. Here, the nucleotide residues within the chain are assumed to be unable to decay (they should be protected therein), whereas those at the end of the chain decay at a rate lower than that of free nucleotides, i.e., *P_NDE_* < *P_ND_*; as for the case concerning amino acids and peptides, likewise, *P_AADE_* < *P_AAD_*. Nucleotides and RNA should be easier to degrade outside protocells (due to the higher water activity), so *F_DO_* > 1; amphiphiles and peptides within a membrane should be protected, so *F_DW_* < *1* (see below for a detailed explanation on the two factors). Because of the self-assembly feature of the membrane, *P_MF_* >> *P_CB_*, *P_AJM_* >> *P_ALM_*, and *P_PJM_* > *P_PLM_*. The movement of molecules should be easier than protocells, so *P_MV_* > *P_MC_*. Other considerations may include: *P_BB_* should be higher than *P_RL_* but lower than *P_NDE_*, *P_PBB_* < *P_AADE_*, *P_APP_*, and *P_AAPP_* > *P_NPP_*, etc.

In consideration of the computational intensity, total materials (*T_NPB,_ T_APB,_* and *T_AAPB_*), “the lower limit number of amphiphiles to form a membrane” (*L_AM_*), and the lengths of the characteristic RNA domains for NSR (*L_NSR_*) and amino acid-binding sites (*L_AABS_*), are set obviously smaller in scale than the corresponding situations in reality. However, such simplifications are believed to be not in conflict with the fundamental rules reflected in the modeling.

In fact, here those parameters concerning the RNA-based protocells were adopted mainly based on our experience in previous studies using similar models [43,45,49,51,52]. When introducing the new parameters concerning amino acids and peptides, a machine learning-like approach was used to automatically explore the parameter values supporting the supposed scenes [59]. The default values listed in Table 1 were adopted to shape the cases for demonstrating our results. Actually, though the outcome of the simulation may be influenced by the change in those “key parameters” (Figure 3) and some of the other parameters (Figure S1 and S2) as explained (Box S1), it turned out to be fairly robust against “moderate adjustments” of most of the parameters.

### 4.3. Detailed Mechanisms Concerning How Some of the Parameters Work

With the breaking of phosphodiester bonds, an RNA molecule may degrade into shorter ones (including nucleotides). When the breaking site of the chain is at a single-chain region, the breaking probability is *P_BB_*. When the breaking site is within a double-chain region, the two parallel bonds may break simultaneously, with the probability of *P_BB_*^2^. However, for the case of outside protocells, a factor is involved (*F_DO_* > 1); the breaking probability for a single-chain is *P_BB_* × *F_DO_*, while that for a double-chain is (*P_BB_* × *F_DO_*)^2^. The factor *F_DO_* also works in the situation of nucleotide decaying and nucleotide residue decaying at the end of an RNA, i.e., *P_ND_*×*F_DO_* and *P_NDE_* × *F_DO_* for the case of outside protocells. Likewise, for the degradation of amphiphiles and the membrane-stabilizing peptide within the protocell membrane, another factor is involved (*F_DW_* < 1), i.e., *P_AD_* × *F_DW_*, *P_AADE_* × *F_DW_*, and *P_PBB_* × *F_DW_* for the case of within the membrane.

The probability of the separation of the two strands of a duplex RNA is actually assumed to be *P_SP_^r^*, where *r* = *n*^1/2^ and *n* is the number of base pairs in the duplex. When *n* = 1, the probability would be *P_SP_*. When *n* increases, the separation of the two strands would be more difficult (because *P_SP_*, as a probability, has a value between 0 and 1). The introduction of the 1/2 corresponds to the consideration that the self-folding of single chains may aid the dissociation of the duplex.

The probability of membrane formation is assumed to be 1 − (1 − *P_MF_*) ^*x*^, where *x* = *a* − *L_AM_* + 1 and *a* is the number of amphiphiles in the grid room. When *a* is equal to *L_AM_* (the lower limit of the number of amphiphiles to form a protocell membrane), the probability of membrane formation is equal to *P_MF_*. This assumption concerns the consideration that the more amphiphiles in a grid room, the more probable they would assemble to form a vesicle.

The probability of an amphiphile leaving the membrane is assumed to be *P_ALM/_*(*y* × *z*), where *y* = 1 + i/(*b*/2)^3/2^ and *z* = 1 + *F_MSP_*×*p*. The item y represents the consideration for the “osmotic pressure effect”: a higher concentration of the inner impermeable ions would cause the protocell to be more swollen, and thus amphiphiles on the membrane are less likely to leave, as suggested by experimental work [60]. Wherein, *i* is the quantity of inner impermeable ions, including nucleotides and RNA (measured by the number of nucleotide residues), and *b* is the quantity of amphiphiles within the membrane. Then, *b*/2 (there are two layers in the membrane) is a “scale” representation of the surface area of the membrane and (*b*/2)^3/2^ is a scale representation of the cellular space. Thus, *i*/(*b*/2)^3/2^ is a representation of the concentration of the ions. The item *z* represents the consideration for the effect of MSP (membrane-stabilizing peptide), in which *p* refers to the number of MSP within the membrane. That is, the desorption rate of amphiphiles from the membrane decreases with the increase of MSP within the membrane, as shown by the experimental work [26]. Note that *F_MSP_* is a parameter which can represent the degree of the MSP effect.

The probability of a nucleotide precursor permeating into a protocell is assumed to be *P_NPP_* × (b/*L_AM_*)/[1 + *i*/(*b*/2)^3/2^], where *i* is the quantity of inner impermeable ions and *b* is the quantity of amphiphiles within the membrane. The element (b/*L_AM_*) represents the consideration of the constraining effect of the cellular space on the influx of nucleotide precursors. That is, when *b* increases, meaning that the cellular space increases correspondingly, the probability of a nucleotide precursor permeating into the protocell would become greater. The introduction of the item *i*/(*b*/2)^3/2^, i.e., the concentration of the inner impermeable ions as explained above, represents the consideration of the effect of Donnan’s equilibrium [61]; simply put, RNA and nucleotides, which are charged and impermeable, may suppress the incoming of permeable materials with the same charge, i.e., nucleotide precursors as it is assumed here (see [51] for a detailed explanation). However, considering that amphiphile precursors and amino acid precursors tend to have no electronic charge, for their permeation, only the influence of the cellular space is considered, that is, the corresponding probabilities are *P_APP_* × (b/*L_AM_*) and *P_AAPP_* × (b/*L_AM_*), respectively.

The probability of protocell division is assumed to be *P_CD_* × (1–2 × *L_AM_*/*b*), where *b* is the quantity of amphiphiles within the membrane. When *b* is no more than twice that of *L_AM_*, the probability is no more than 0, i.e., the protocell could not divide. This assumption considers the fact that the larger the protocell, the more probable it would divide, on account of the physical instability.

The probability of the movement of an RNA molecule is assumed to be *P_MV_*/*m*^1/2^, where *m* is the mass of the RNA, relative to a nucleotide. This assumption represents the consideration of the effect of the molecular size on the molecular movement. The square root was adopted here according to the Zimm model, concerning the diffusion coefficient of the polymer molecules in the solution [62]. Likewise, the probability of the movement of a peptide is also assumed to be *P_MV_*/*m*^1/2^, where *m* is the mass of the peptide relative to an amino acid. Note that when there are amino acids or peptides binding on an RNA, the total mass would be calculated into *m* in regard to the movement of the complex. 

(Note: Source codes of the simulation program in C language can be obtained from GitHub—see Data Availability Statement. Besides the role of evidencing the reproducibility of the present study, the source codes present more details about the implementation of the model and may help readers to understand the simulation better).

## Figures and Tables

**Table 1:** Parameters used in the computer simulation.

Probabilities	Descriptions	Values
*P_AD_*	An amphiphile decaying into its precursor	0.01
*P_AF_*	An amphiphile forming from its precursor	0.02
*P_AJM_*	An amphiphile joining the membrane	0.2
*P_ALM_*	An amphiphile leaving the membrane	0.001
*P_APP_*	An amphiphile precursor permeating through the membrane	0.9
*P_AT_*	An RNA template attracting a substrate (by base-pairing)	0.9
*P_BB_*	A phosphodiester bond breaking within an RNA chain	1 × 10^−5^
*P_CB_*	A protocell breaking	2 × 10^−4^
*P_CD_*	A protocell dividing	0.05
*P_CF_*	Two adjacent protocells fusing with each other	0.001
*P_FP_*	The false base-pairing when a template attracts a substrate	1 × 10^−4^
*P_MC_*	A protocell moving	0.1
*P_MF_*	A membrane forming	0.1
*P_MV_*	A nucleotide/amphiphile/amino acid (or its precursor) moving	0.9
*P_ND_*	A nucleotide decaying into its precursor	0.05
*P_NDE_*	A nucleotide residue decaying at an RNA’s chain end	0.001
*P_NF_*	A nucleotide forming from its precursor (non-enzymatic)	0.02
*P_NFR_*	A nucleotide forming from its precursor catalyzed by NSR	0.5
*P_NPP_*	A nucleotide precursor permeating through the membrane	0.5
*P_RL_*	The Random ligation of nucleotides and RNA	1 × 10^−6^
*P_SP_*	The separation of a base pair	0.5
*P_TL_*	The template-directed ligation of RNA	0.5
*P_AABR_*	An amino acid binding onto an RNA template	0.9
*P*_AAD_	An amino acid decaying into its precursor	0.2
*P_AADE_*	An amino acid residue decaying at a peptide’s chain end	0.1
*P_AAF_*	An amino acid forming from its precursor	0.1
*P_AAPP_*	An amino acid precursor permeating through the membrane	0.9
*P_AATL_*	Amino acids’ ligation on an RNA template (DRT mechanism)	0.5
*P_NFP_*	A nucleotide forming from its precursor catalyzed by NSP	0.5
*P_PBB_*	A peptide bond breaking	0.01
*P_PJM_*	A peptide joining the membrane	0.9
*P_PLM_*	A peptide leaving the membrane	0.1
*P_PLR_*	An amino acid or peptide leaving RNA	0.2
**Others**	**Descriptions**	**Values**
*N*	The system is defined as an *N* × *N* grid	30
*T_NPB_*	Total nucleotide precursors introduced in the beginning	50,000
*T_APB_*	Total amphiphile precursors introduced in the beginning	50,000
*T_AAPB_*	Total amino acid precursors introduced in the beginning	50,000
*F_DO_*	The factor of molecular degradation outside protocells	20
*F_DW_*	The factor of molecular degradation within the membrane	0.1
*F_MSP_*	The factor concerning the membrane-stabilizing peptide	1
*L_AM_*	The lower limit number of amphiphiles to form a membrane	200
*L_NSR_*	The length of characteristic sequence of NSR (in nucleotides)	10
*L_AABS_*	The length of amino acid-binding sites (in nucleotides)	5

Note: The upper portion of the probabilities (above the dashed line) include those for RNA-based protocells (derived from our previous work; with names in alphabetical order), whereas the lower portion of the probabilities are those concerning amino acids, peptides, and the DRT mechanism (with names in alphabetical order). The simulation cases shown in this paper adopt the values listed here (as default values), unless being stated explicitly to be different.

## Data Availability

The source code of the approach can be obtained from:https://github.com/mwt2001gh/DRT-Membrane-stabilizing-peptide/blob/main/MSP-Fig4a.cpp. The version corresponds the case in Figure 4a (accessed on 29 December 2022).

## Supplementary Materials

The following supporting information can be downloaded at: https://www.mdpi.com/article/10.3390/life13020523/s1. **Figure S1.** The influence of other parameters (in addition to those shown in Figure 3) on the spread of MSPG protocells (part 1). The representations are the same as those in Figure 3. For a clearer appearance, here no italic and subscript has been applied in the names of the parameters. The values adopted at the three critical turning points are listed in Table S1. See Box S1 for a comment on the influence. **Figure S2.** The influence of other parameters (in addition to those shown in Figure 3) on the spread of MSPG protocells (part 2). This is a continuation of Figure S1. The parameter values adopted at the three critical turning points are listed in Table S1. See Box S1 for a comment on the influence. **Figure S3.** The effect of “function-lagging” can be reflected from the influences of *P_PBB_* and *P_AADE_* on the spread of MSPG protocells. The representations are the same as those in Figure 3. For the upper two subfigures, the default value of *P_AADE_* is 0.01 and that of *P_PBB_* is 0.1. In the subfigure *P_AADE_*’, when turned up, the parameter is changed to 0.02, 0.05, and 0.1 at the three turning steps, respectively, and when turned down, is changed to 0.005, 0.002, and 0.001, respectively. In the subfigure *P_PBB_*’, when turned up, the parameter is changed to 0.2, 0.5, and 0.9, and when turned down, is changed to 0.05, 0.02, and 0.01. Obviously, here the effective one is *P_PBB_* and the turning down of *P_AADE_* has little influence. However, for the lower two subfigures, the default values of *P_AADE_* is 0.1 and that of *P_PBB_* is 0.01, then the effective one is *P_AADE_*, and the turning down of *P_PBB_* has little influence (the subfigure *P_AADE_* is identical to the one in Figure 3 and the subfigure *P_PBB_* is identical to the one in Figure S1, and they are placed here for a straightforward comparison with the upper two subfigures). **Figure S4.** The competing ribozyme and peptide gene with the same function (NSR and NSPG) are difficult to co-spread. For all the cases, at step 1 × 10^4^, ten protocells each containing five copies of NSR, a control RNA, NSPG, and a control peptide gene are inoculated into the system. For all the cases, the efficiency of NSR is fixed (*P_NFR_* = 0.5). The efficiency of NSP, *P_NFP_* adopts different values as shown in the subfigures (a, b, c, and d). **Table S1**. The values adopted in the parameter analysis (Figures S1 and S2). **Box S1.** On the influence of parameters (Figures S1 and S2).
