# Supplementary material for: Towards an RNA/Peptides World by the Direct RNA Template Mechanism: The Emergence of Membrane-Stabilizing Peptides in RNA-Based Protocells
Source: Life (Basel). 2023 Feb 14;13(2):523. doi: 10.3390/life13020523 (PMC9966593; doi:10.3390/life13020523)
Supplement: Supplementary file 1 [file life-13-00523-s001.zip › Box_S1.pdf]

Box S1. On the influence of parameters (Figure S1 and S2)

Firstly, we note that the default values of the parameters we adopted are almost the best for the spread of protocells containing MSPG (filled blue circles) – that is, the change of parameter values, either upwards or downwards, could barely improve the level of the MSPG protocells (orange open circles for "upwards" and cyan open circles for "downwards"). This means our initial parameter-exploration is quite successful. Secondly, we see that the spread of MSPG protocells is robust to the change of most parameter values. In the analysis, when turning up or down a parameter, a scale of 2 or 2.5 times was adopted, unless the probability might be larger than 1 (see Table S1 for details). That is, for many parameters the range of the value change is 100 times as a whole (for the cases that the probability might be larger than 1, at least 10 times) – however, in nearly 2/3 cases, the influence on the spread of MSPG protocells is little or quite limited. Thirdly, we comment briefly as follows on the cases in which the parameter change brings about obvious influence.

A high probability of peptide leaving the membrane (P_{PLM}) is unfavorable because MSPG would not play its role outside the membrane. A high error rate in the replication of RNA (P_{FP}) is unfavorable because the heredity of MSPG is weakened. A low probability for the separation of a base pair (P_{SP}) is unfavorable because of the difficulty of strand separation in the RNA replication. Both a low probability of amphiphiles joining the membrane (P_{AJM}) and a low probability of amphiphiles leaving the membrane (P_{ALM}) are unfavorable because the effect of MSP is weakened (see Figure 1). A low probability of amphiphile-forming (P_{AF}) or a high probability of amphiphile-decaying (P_{AD}) is unfavorable because the amphiphiles are insufficient to support the growth or even the existence of protocells. A large factor of molecular degradation within the membrane (F_{DW}) is unfavorable because both amphiphiles within the membrane and MSP within the membrane tend to degrade. A small factor of molecular degradation outside the protocells (F_{DO}) would be unfavorable because the superiority of protocells is weakened. A high probability of protocell-fusing (P_{CF}) is unfavorable because the protocells without MSPG tend to fuse with those containing MSPG and thus the MSPG is "parasitized". Related to this, a high moving rate of protocells (P_{MC}) is unfavorable because this would tend to bring the protocells without MSPG adjacent to the MSPG protocells and enhance the likelihood of the cell-fusion. A high probability of protocell-breaking (P_{CB}) is unfavorable because the existence of protocells becomes problematic.
