# Supplementary material for: Towards an RNA/Peptides World by the Direct RNA Template Mechanism: The Emergence of Membrane-Stabilizing Peptides in RNA-Based Protocells
Source: Life (Basel). 2023 Feb 14;13(2):523. doi: 10.3390/life13020523 (PMC9966593; doi:10.3390/life13020523)
Supplement: Supplementary file 1 [file life-13-00523-s001.zip › Table_S1.pdf]

**Table S1. The values adopted in the parameter analysis (Figure S1 and S2)**

| Up-v3 | Up-v2 | Up-v1 | v0 | Down-v1 | Down-v2 | Down-v3 |
|---|---|---|---|---|---|---|
| 0.99 | 0.98 | 0.95 | $P_{PJM}$=0.9 | 0.5 | 0.2 | 0.1 |
| 0.9 | 0.5 | 0.2 | $P_{PLM}$=0.1 | 0.05 | 0.02 | 0.01 |
| 0.9 | 0.5 | 0.2 | $P_{AAF}$=0.1 | 0.05 | 0.02 | 0.01 |
| 0.95 | 0.9 | 0.5 | $P_{AAD}$=0.2 | 0.1 | 0.05 | 0.02 |
| 0.95 | 0.9 | 0.5 | $P_{PLR}$=0.2 | 0.1 | 0.05 | 0.02 |
| 0.1 | 0.05 | 0.02 | $P_{PBB}$=0.01 | 0.005 | 0.002 | 0.001 |
| 0.2 | 0.1 | 0.05 | $P_{NF}$=0.02 | 0.01 | 0.005 | 0.002 |
| 0.5 | 0.2 | 0.1 | $P_{ND}$=0.05 | 0.02 | 0.01 | 0.005 |
| 0.01 | 0.005 | 0.002 | $P_{NDE}$=0.001 | $5\times10^{-4}$ | $2\times10^{-4}$ | $1\times10^{-4}$ |
| $1\times10^{-4}$ | $5\times10^{-5}$ | $2\times10^{-5}$ | $P_{BB}=1\times10^{-5}$ | $5\times10^{-6}$ | $2\times10^{-6}$ | $1\times10^{-6}$ |
| 0.001 | $5\times10^{-4}$ | $2\times10^{-4}$ | $P_{FP}=1\times10^{-4}$ | $5\times10^{-5}$ | $2\times10^{-5}$ | $1\times10^{-5}$ |
| 0.98 | 0.95 | 0.9 | $P_{SP}$=0.5 | 0.2 | 0.1 | 0.05 |
| 0.99 | 0.98 | 0.95 | $P_{AT}$=0.9 | 0.5 | 0.2 | 0.1 |
| 0.98 | 0.95 | 0.9 | $P_{TL}$=0.5 | 0.2 | 0.1 | 0.05 |
| $1\times10^{-5}$ | $5\times10^{-6}$ | $2\times10^{-6}$ | $P_{RL}=1\times10^{-6}$ | $5\times10^{-7}$ | $2\times10^{-7}$ | $1\times10^{-7}$ |
| 0.95 | 0.9 | 0.5 | $P_{AJM}$=0.2 | 0.1 | 0.05 | 0.02 |
| 0.01 | 0.005 | 0.002 | $P_{ALM}$=0.001 | $5\times10^{-4}$ | $2\times10^{-4}$ | $1\times10^{-4}$ |
| 0.2 | 0.1 | 0.05 | $P_{AF}$=0.02 | 0.01 | 0.005 | 0.002 |
| 0.1 | 0.05 | 0.02 | $P_{AD}$=0.01 | 0.005 | 0.002 | 0.001 |
| 0.9 | 0.5 | 0.2 | $F_{DW}$=0.1 | 0.05 | 0.02 | 0.01 |
| 0.99 | 0.98 | 0.95 | $P_{APP}$=0.9 | 0.5 | 0.2 | 0.1 |
| 0.98 | 0.95 | 0.9 | $P_{NPP}$=0.5 | 0.2 | 0.1 | 0.05 |
| 0.99 | 0.98 | 0.95 | $P_{AAPP}$=0.9 | 0.5 | 0.2 | 0.1 |
| 0.99 | 0.98 | 0.95 | $P_{MV}$=0.9 | 0.5 | 0.2 | 0.1 |
| 200 | 100 | 50 | $F_{DO}$=20 | 10 | 5 | 2 |
| 0.01 | 0.005 | 0.002 | $P_{CF}$=0.001 | $5\times10^{-4}$ | $2\times10^{-4}$ | $1\times10^{-4}$ |
| 0.5 | 0.2 | 0.1 | $P_{CD}$=0.05 | 0.02 | 0.01 | 0.005 |
| $2\times10^{-3}$ | $1\times10^{-3}$ | $5\times10^{-4}$ | $P_{CB}=2\times10^{-4}$ | $1\times10^{-4}$ | $5\times10^{-5}$ | $2\times10^{-5}$ |
| 0.9 | 0.5 | 0.2 | $P_{MC}$=0.1 | 0.05 | 0.02 | 0.01 |
| 0.9 | 0.5 | 0.2 | $P_{MF}$=0.1 | 0.05 | 0.02 | 0.01 |

Note: The upper portion of the probabilities (above the dashed line) is for Figure S1 and the lower portion is for Figure S2. "v0" means the default value; "Up-v1", "Up-v2" and "Up-v3" means the values adopted at the three turning steps (one after another; see red arrows in the figures) for the case of parameter-turning-up; "Down-v1", "Down-v2" and "Down-v3" means the values adopted at the three turning steps for the case of parameter-turning-down.
