# Supplementary figures and images for: Towards an RNA/Peptides World by the Direct RNA Template Mechanism: The Emergence of Membrane-Stabilizing Peptides in RNA-Based Protocells
Source: Life (Basel). 2023 Feb 14;13(2):523. doi: 10.3390/life13020523 (PMC9966593; doi:10.3390/life13020523)

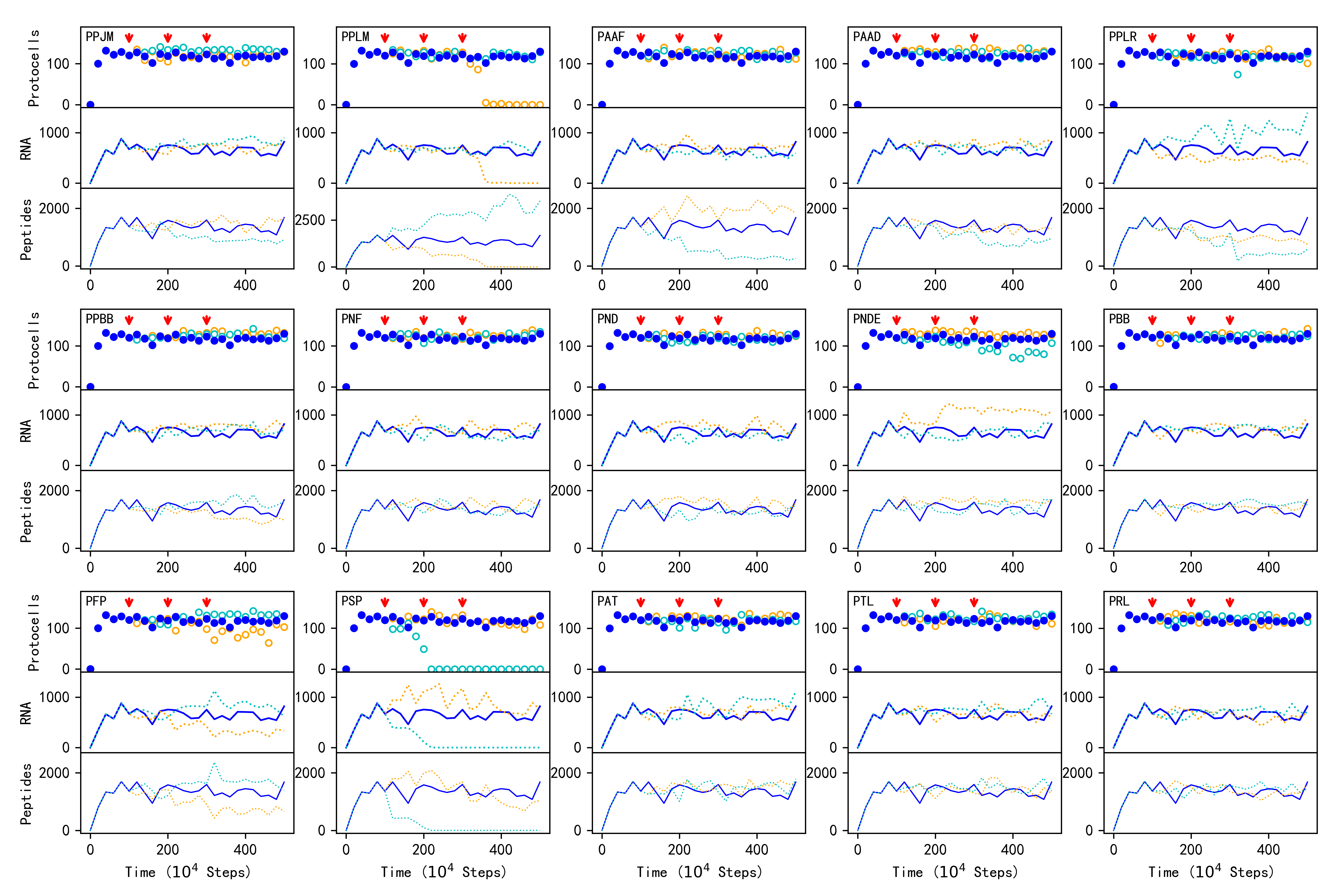

Supplement: Supplementary file 1 [file life-13-00523-s001.zip › Fig_S1.png]

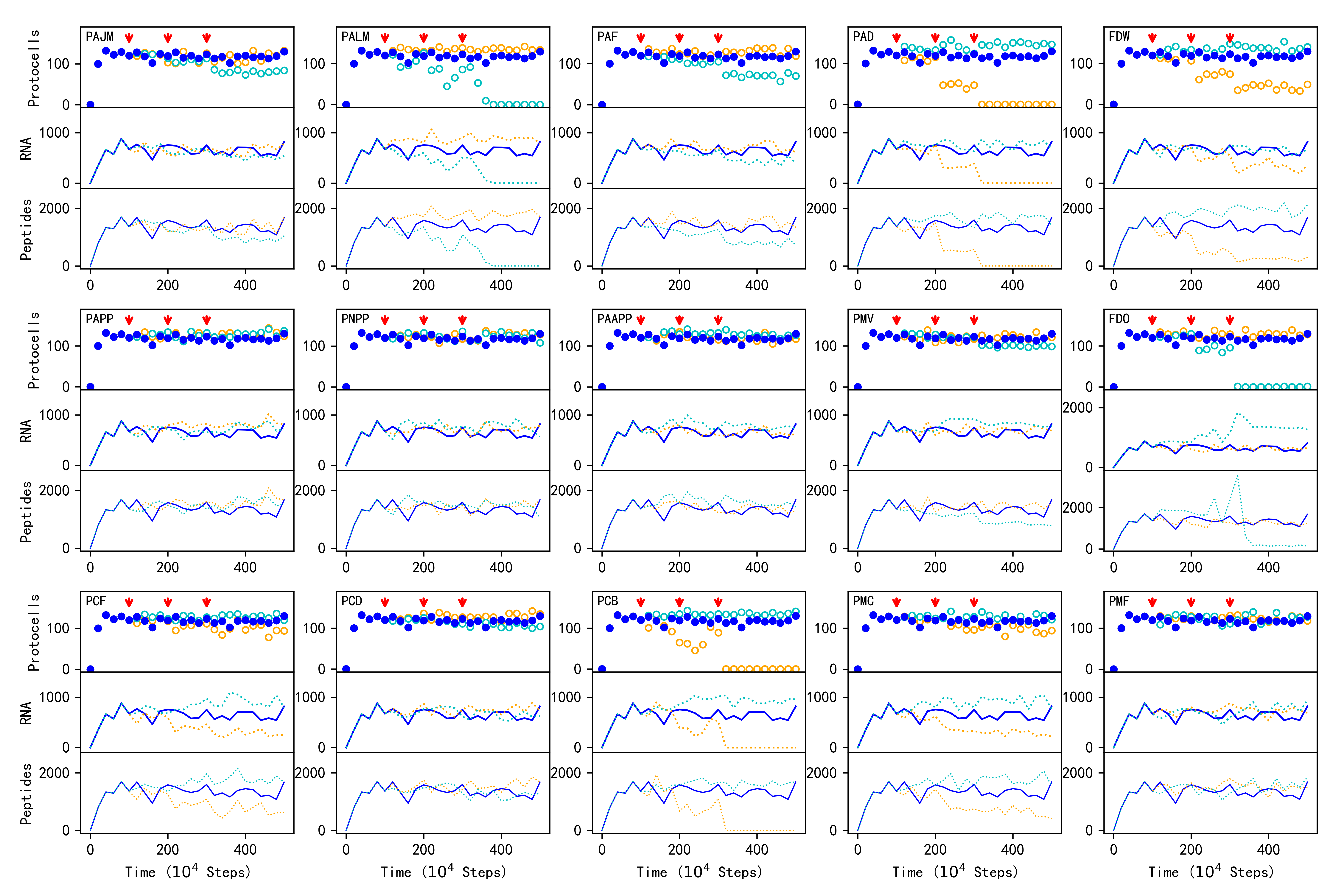

Supplement: Supplementary file 1 [file life-13-00523-s001.zip › Fig_S2.png]

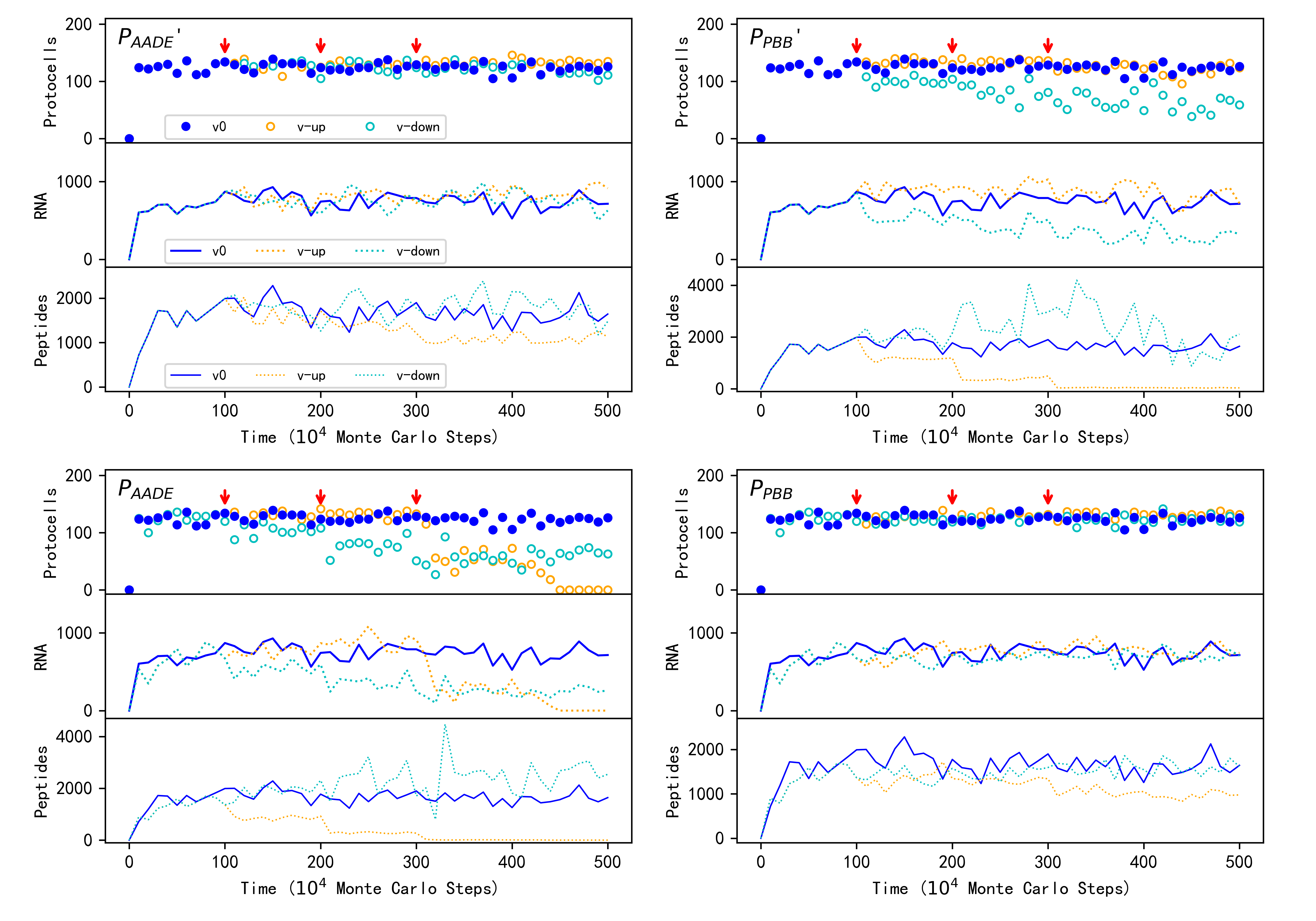

Supplement: Supplementary file 1 [file life-13-00523-s001.zip › Fig_S3.png]

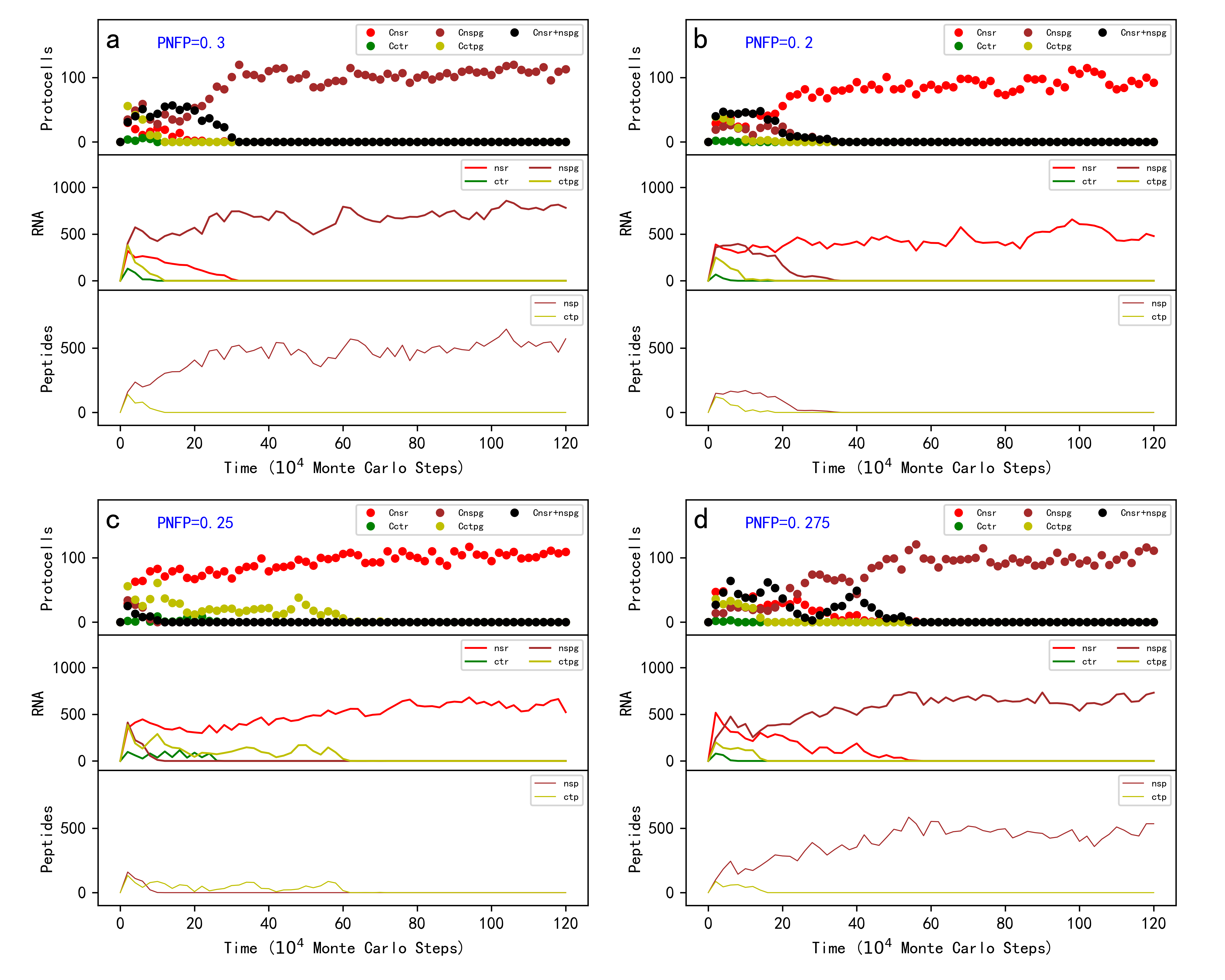

Supplement: Supplementary file 1 [file life-13-00523-s001.zip › Fig_S4.png]
